# Synergistic Anticancer Activity of Plumbagin and Xanthohumol Combination on Pancreatic Cancer Models

**DOI:** 10.3390/ijms25042340

**Published:** 2024-02-16

**Authors:** Ranjith Palanisamy, Nimnaka Indrajith Kahingalage, David Archibald, Ilaria Casari, Marco Falasca

**Affiliations:** 1 Metabolic Signalling Group, Curtin Medical School, Curtin Health Innovation Research Institute, Curtin University, Perth 6102, Australia; ranjith.palanisamy@telethonkids.org.au (R.P.); indrajithnimnaka7@gmail.com (N.I.K.); ilacasari@gmail.com (I.C.); 2 Backreef Oil Pty Ltd., Perth 6015, Australia; david.archibald@westnet.com.au; 3 Department of Medicine and Surgery, University of Parma, Via Volturno 39, 43125 Parma, Italy

**Keywords:** pancreatic cancer, plumbagin, xanthohumol, drug combinations, phytochemicals

## Abstract

Among diverse cancers, pancreatic cancer is one of the most aggressive types due to inadequate diagnostic options and treatments available. Therefore, there is a necessity to use combination chemotherapy options to overcome the chemoresistance of pancreatic cancer cells. Plumbagin and xanthohumol, natural compounds isolated from the Plumbaginaceae family and *Humulus lupulus,* respectively, have been used to treat various cancers. In this study, we investigated the anticancer effects of a combination of plumbagin and xanthohumol on pancreatic cancer models, as well as the underlying mechanism. We have screened in vitro numerous plant-derived extracts and compounds and tested in vivo the most effective combination, plumbagin and xanthohumol, using a transgenic model of pancreatic cancer KPC (KrasLSL.G12D/+; p53R172H/+; PdxCretg/+). A significant synergistic anticancer activity of plumbagin and xanthohumol combinations on different pancreatic cancer cell lines was found. The combination treatment of plumbagin and xanthohumol influences the levels of B-cell lymphoma (BCL2), which are known to be associated with apoptosis in both cell lysates and tissues. More importantly, the survival of a transgenic mouse model of pancreatic cancer KPC treated with a combination of plumbagin and xanthohumol was significantly increased, and the effect on BCL2 levels has been confirmed. These results provide a foundation for a potential new treatment for pancreatic cancer based on plumbagin and xanthohumol combinations.

## 1. Introduction

Pancreatic cancer (PC) is one of the most hostile and intractable types of cancer in the world with an incidence-to-mortality ratio of close to one. It has an extremely poor prognosis resulting in a five-year survival rate of approximately 12% [1,2]. Pancreatic cancer is estimated to become the second most common cause of cancer-related death in 2040 [3]. Surgery is the only possible treatment for primary-stage or resectable pancreatic tumours [4] and, even in this case, cancer relapses and metastasis development are a major concern [5]. Presently, chemotherapy is the only feasible option available to inoperable patients, and gemcitabine combined with albumin-bound paclitaxel is the standard first-line treatment [6]. For cancer metastatic patients who can tolerate it, FOLFIRINOX, a combination agent, is the treatment of preference [7]. However, the lack of effectiveness due to the high chemoresistance of PC, high toxicity, and the wide variety of side effects of chemotherapy demand a search for new lines of treatment [4,8]. The benefits of a diet rich in fruit and vegetables and its role in preventing numerous types of cancer have become common knowledge in the last few decades [9]. Consequently, many researchers are currently focusing on natural plant extracts and their isolated compounds as a treatment for pancreatic and other cancer types [10]. Dietary-derived anti-cancer agents are divided into blocking agents and suppressing agents, depending on their effect as chemopreventive agents throughout the various stages of the carcinogenic process [11,12]. Examples of phytochemicals contained in food and vegetables that battle cancer include cancer-blocking agents such as sulforaphane, the natural compound found in broccoli, and suppressing agents such as 6–gingerol, found in ginger, and capsaicin, found in the chilli pepper [13,14]. Some natural plant extracts have been identified as effective anticancer agents and have reached the clinical trial stage [15,16]. Polyphenols, and in particular flavonoids, are biologically active molecules present in many food plants. As well as having antioxidant activity, they are known to down-regulate the production of antiapoptotic proteins in the apoptotic cascade of the caspases [17]. The flavonoid xanthohumol (XH) has been found to inhibit cell proliferation and to sensitise cells to chemotherapy in in vitro studies on colorectal cancer cells [18,19]. Interestingly, XH has been found to have antitumour activity in different cancer cell lines including pancreatic cancer cell lines [20,21,22]. Similarly, plumbagin (PL), a plant-derived naphthoquinone, has been shown to have anticancer activity in vitro and in vivo [22,23].

The objectives of this study were to test the potential anticancer activity of different plant extracts and plant-derived bioactive compounds in pancreatic cancer. The most promising compounds were selected and tested in combination on a panel of different pancreatic human cell lines. Studies on the possible mechanisms of action of these compounds were also undertaken. As protein signalling is a mechanism in the progression of cancer, various protein signalling pathways were examined to determine which protein pathways were affected by the extracts or compounds. Two plant-based agents, PL and XH, were selected after the initial screening thanks to their enhanced action on cancer cell growth and proliferation. In the present study, we present data showing the anticancer activity of PL and XH combinations used as a treatment in vitro and substantiated in a transgenic mouse model of pancreatic cancer.

## 2. Results

### 2.1. In Vitro Screening

A variety of plant extracts and compounds were tested on a panel of human pancreatic (HPAF-II, AsPC-1) and mouse pancreatic (mT4-2D and KPC) cancer cell lines. The IC_50_ of each different agent was calculated after dose-response experiments were performed (Table 1). The most interesting compound studied was PL, which showed IC_50_ values on human pancreatic cancer cell lines HPAF-II and AsPC-1 of 1.33 and 0.98 µg/mL, respectively (Table 1). PL was even more effective when tested on human AsPC-1 tumourspheres, showing increased activity with a lower IC_50_ compared to parental AsPC-1 cancer cells of 0.53 µg/mL. When tested on two transgenic pancreatic mouse-derived cancer cell lines, mT4-2D and KPC, cell lines derived from KPC organoid cultures and KPC mice, respectively, IC_50_ values of 0.4 and 0.58 µg/mL, respectively, were observed (Table 1). Subsequently, PL was tested on the human ovarian cancer cell line A2780, the human prostate cancer cell line PC3, and the breast adenocarcinoma syngeneic cell line 4T1, obtaining IC_50_ values of 0.56, 0.93, and 0.74 µg/mL, respectively (Table 1). In addition, IC_50_ of XH on HPAF-II and AsPC-1 were recorded as 3.89 µg/mL and 6.8 µg/mL, respectively. An IC_50_ of 9.15 µg/mL was recorded treating mT4-2D cells with XH.

Other cancer cell lines, A2780, PC3, and 4T1, expressed IC_50_ values of 0.3478 µg/mL, 4.968 µg/mL, and 1.26 µg/mL, respectively, after treatments with XH. As observed with PL, when XH was tested on Human AsPC-1 tumour spheres, it showed an increased activity with a lower IC_50_ compared to parental AsPC-1 cancer cells of 1.3 µg/mL. Amongst the extracts, ginger and hops were demonstrated to be the most active. Sulforaphane, 6-shogaol, and 6-gingerol showed an interesting effect, with sulforaphane being by far the most effective agent. On the contrary, broccoli seed and broccoli seed combined with chilli extracts did show very little activity on all cell lines tested, with the IC_50_ values of these extracts being quite high. Peppermint and marjoram demonstrated to have modest activity with an IC_50_ above 40, and pepper performed slightly better (IC_50_ above 20 µg/mL). The active compound of ginger, zingerone, has only mild cytotoxicity on ASPC-1 cells (IC_50_ 37.4 µg/mL); piperine and capsaicin were fairly active on those cells (IC_50_ 13.2 µg/mL and 15.3 µg/mL, respectively), but capsaicin was more active on HPAFII cells (IC_50_ 6.1 µg/mL).

### 2.2. In Vitro Combination Studies

**Figure 1:**
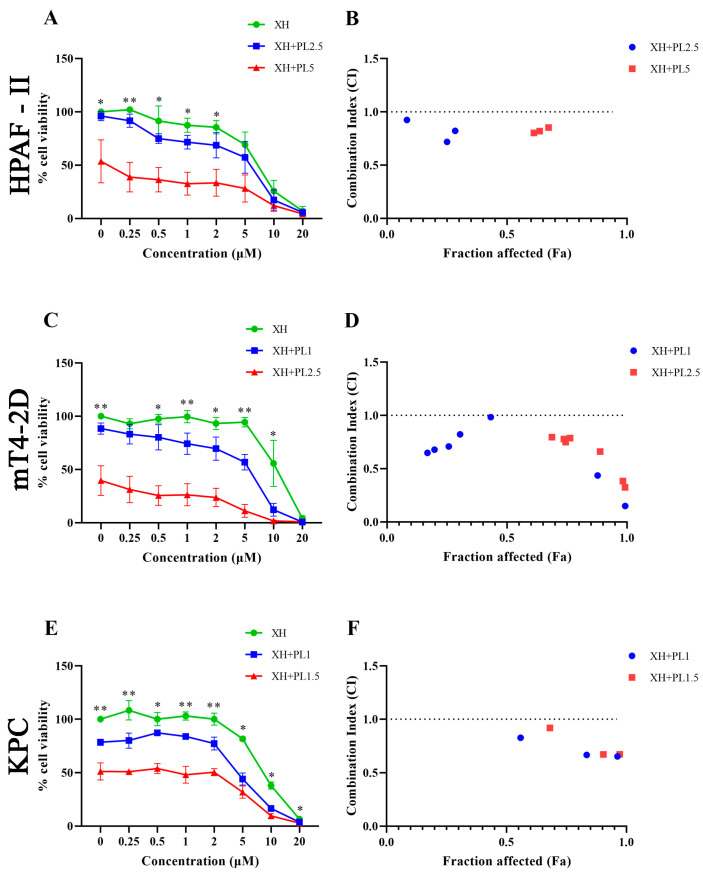
Synergistic effects of XH and PL on PDAC cell lines. (**A**,**C**,**E**) The viability of the cells treated with XH combined with PL was detected with dose-response MTT assays. KPC, Mt4-2D, and HPAF-II cells were grown and treated with XH or PL alone, or XH combined with PL, at two different concentrations for 72 h. Each experiment was repeated three times independently. Data are shown as means ± S.E and were analysed with one-way ANOVA. * *p* 0.05, ** *p* < 0.01 vs. 0 µg/mL. (**B**,**D**,**F**) Combination Index (CI)-effect plot. The CI values were calculated using the CompuSyn program to determine the combined effects of XH and PL in PDAC cell lines. The combinations were synergistic when CI values were < 1. The data are the mean values from three independent experiments. XH—Xanthohumol; PL—Plumbagin.

After having screened the extracts and compounds as single agents, the most effective ones were selected and tested again in various combinations to assess their anticancer potential and possible synergistic effects. The MTT assay was used to examine the potential synergistic effect of extract and compound combinations which were tested on HPAF-II, Mt4-2D, and KPC pancreatic cancer cell lines. The combination of PL/XH was found to be the most active. According to the Chou–Talalay method [24], a Combination Index (CI) value of less than 1 can be considered as synergism, while CI = 1 represents an additive effect and CI > 1 is antagonistic. Interestingly, we observed synergistic effects (CI < 1) in every concentration of XH combined with a concentration close to IC_50_ of PL on Mt4-2D cells. Effective synergism was observed when XH concentrations were combined with a concentration close to IC_25_ of PL on HPAF-II, Mt4-2D, and KPC cells (Figure 1). All of these results were consistent with the MTT assay reading obtained after combination treatments (Figure 1). The CompuSyn analysis gave a Combination Index (CI) consistent with MTT assay readings, showing a synergistic effect at low concentrations on HPAF-II (PL 2.5 and 5 µM, XH 0.25, 0.5, and 1 µg/mL) (Figure 1). Additionally, a synergistic effect was observed when the Mt4-2D cell line was treated with low concentrations of both PL and XH (PL 1 and 2.5 µM, XH 0.25, 0.5, 1, 2.5, and 5 µg/mL) (Figure 1). On the KPC cell line, the synergistic effect was observed using concentrations of 1 and 1.5 µM for PL and a higher concentration of 5, 10, and 20 µg/mL for XH (Figure 1). Hence, it can be clearly seen that PL and XH combination treatments are more effective than individual treatments in our in vitro studies.

### 2.3. Plumbagin and Xanthohumol Target BCL2 on HPAF-II and mT4-2D Pancreatic Cancer Cell Lines

**Figure 2:**
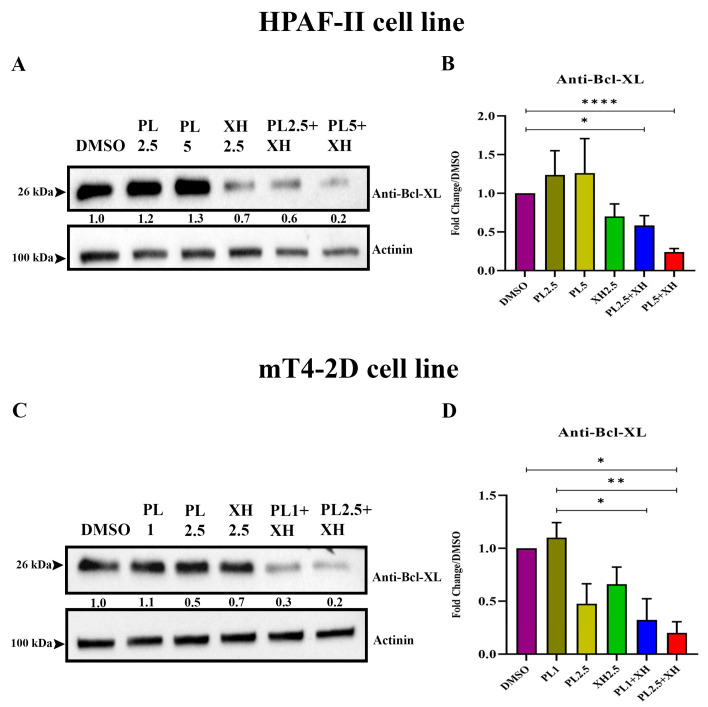
(**A**) Western blot image showing HPAF-II pancreatic cancer cell line after treatment with DMSO, PL (2.5 and 5 µM), XH (2.5 µg/mL), and combinations of XH 2.5 µg/mL with PL 2.5 and 5 µM concentrations. (**B**) Quantification of protein bands (BCL2) in HPAF-II cells analysed using the Image Lab 5.1 software. (**C**) Western blot image showing mT4-2D pancreatic cancer cell line after treatment with DMSO, PL (1 and 2.5 µM), XH (2.5 µg/mL), and combinations of XH 2.5 µg/mL with PL 1 and 2.5 µM concentrations. (**D**) Quantification of protein bands (BCL2) in mT4-2D cells analysed using the Image Lab 5.1 software. All experiments are presented as mean ± SEM of 3 independent experiments. Unpaired two-tailed student’s *t*-test and GraphPad Prism version 9.4.1 were used for statistical analysis, * *p* < 0.01, ** *p* < 0.001, **** *p* < 0.0001.

To gain an insight into the mechanism of action of the PL/XH combination, western blot analysis was performed to investigate signalling pathways involved in cancer development. HPAF-II and mT4-2D pancreatic cells were treated for 24 h with PL and XH alone and in two different combination concentrations of PL and XH (PL 1, 2.5, and 5 µM, XH at 2.5 µg/mL), as shown in Figure 2. Western blot analysis shows that combined concentrations of PL and XH in both cell lines decreased the level of the BCL2 (B-cell lymphoma 2, *BCL2*) protein compared to both the DMSO control and individual concentrations (Figure 2A–D), with the combination PL at 5 µM plus XH at 2.5 µg/mL being the most effective in HPAF-II cells (Figure 2B), while the combination treatments PL 1 and 2.5 µM plus XH at 2.5 µg/mL on mT4-2D cells reduced the level of BCL2 in a statistically significant way (Figure 2D). CXCR4 (C-X-C Motif Chemokine Receptor 4, *CXCR4*) and phospho-Akt 473 (AKT serine/threonine kinase 1, *AKT1*) levels were also tested in HPAF and mT4-2D cells upon 24 h treatment with a combination of PL and XH, but the treatments did not give consistent results (Supplementary Figure S1).

### 2.4. In Vivo Studies

**Figure 3:**
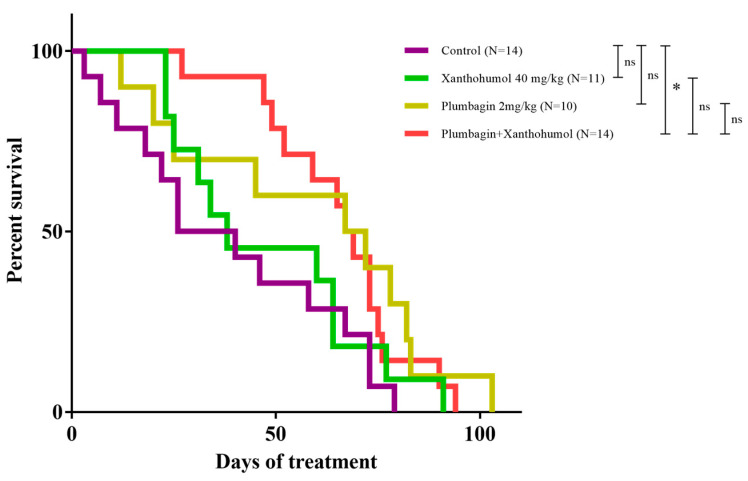
Kaplan–Meier survival curve of KPC mice treated with vehicle (*n* = 14), xanthohumol (*n* = 11), plumbagin (*n* = 10), and xanthohumol plus plumbagin (*n* = 14). Logrank (Mantel–Cox) test *p* = 0.0845, Logrank test for trend *p* = 0.0228, Gehan–Breslow–Wilcoxon test *p* = 0.0484. The xanthohumol plus plumbagin was the only treatment group to show a significant difference in the survival of KPC mice when compared to the control. Logrank test (Mantel–Cox) *p* = 0.0300, Gehan–Breslow–Wilcoxon test *p* = 0.0086; * *p* < 0.05. Curves indicate days after the start of each treatment.

To assess the anticancer activity of PL in vivo, a KPC transgenic mouse model of pancreatic cancer was employed. This model is characterised by KrasLSL.G12D/+, p53R172H/+, and PdxCretg/+ mutations, and it is designed to spontaneously develop PDAC in a way that strongly mimics the human disease, both histologically and pathologically [25]. PL was combined with XH to evaluate the effects of this combination treatment in the KPC mice model. Once tumour development was established by palpation, mice were randomly divided into a control group and three treatment groups, composed of 10–14 mice. The four groups were then treated daily by intraperitoneal injection with vehicle only, 2 mg/kg of PL, 40 mg/kg of XH, and a combination of 2 mg/kg of PL and 40 mg/kg of XH. The treatment with PL resulted in increased survival of the mice belonging to the PL group (*p* = 0.0628) (Figure 3). No difference in survival was observed in mice treated with XH alone. Interestingly, a statistically significant increase (*p* = 0.0300) in the survival of mice treated with the PL/XH combination was observed (Figure 3). The weight of the pancreas extracted from all the mice also showed a significant reduction in the XH/PL combination compared to the control (Supplementary Figure S2).

**Figure 4:**
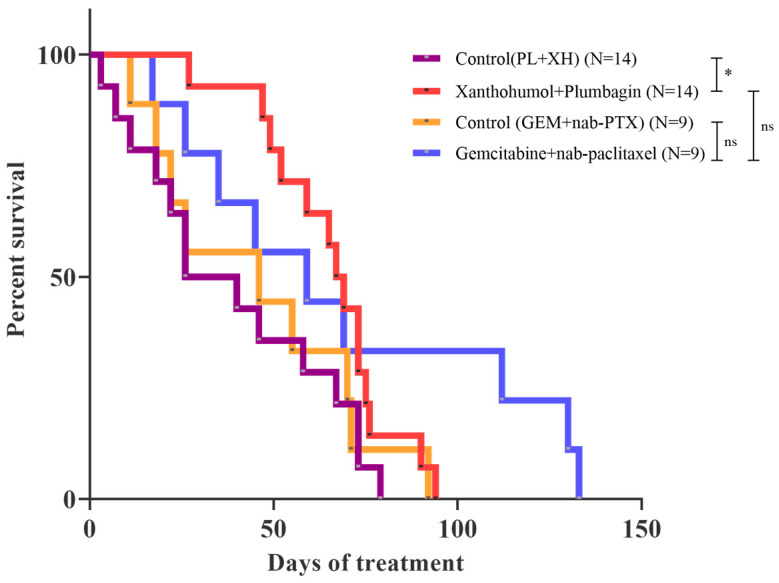
Kaplan–Meier survival curve showing KPC mice survival of xanthohumol plus plumbagin treatment compared against gemcitabine and nab-paclitaxel combination with their respective controls. Curves indicate days after the start of each treatment. Logrank (Mantel–Cox) test *p* = 0.0626, Logrank test for trend *p* = 0.0639, Gehan–Breslow–Wilcoxon test *p* = 0.0535; * *p* < 0.05. The curves control (XH/PL) and xanthohumol plus plumbagin are the same as presented in Figure 3.

Having established the existence of an anticancer activity (prolonged median survival) of the PL/XH combination in KPC mice, we proceeded to compare it to the standard chemotherapy treatment for PDAC, a gemcitabine plus albumin-bound paclitaxel (nab-paclitaxel) combination. A group of nine mice was administered intravenously with 50 mg/kg of gemcitabine plus 6.25 mg/kg of nab-paclitaxel (on days 1, 8, and 15 of each 28-day treatment cycle), while the nine mice in the control group received only vehicle. The ratio and schedule of the two drugs used are the same used in the treatment of pancreatic cancer patients. Interestingly, only three mice out of nine (33%) responded to the gemcitabine plus nab-paclitaxel combination, and the overall chemotherapy treatment was not statistically significant (Figure 4). In addition, the PL/XH combination had better median survival than the chemotherapy treatment and a twofold increase compared to the control group (Supplementary Figure S3). To test the effect of different treatments on their weight, the KPC mice were weighed every day and at the end of the experiment. The recorded data are presented in Supplementary Figure S4, which shows that all treatments did not lead to any significant weight loss. Weight loss was noted in the KPC mice when the administration of PL or the XH/PL combination was started, but the weight loss on average was around 5% among the mice in those treatment groups. The weights plateaued out around 2 weeks into treatment and the combination group gained some weight along the period compared to the plumbagin group.

### 2.5. Plumbagin and Xanthohumol Target BCL2 and pSTAT3 on Mice Tissue

**Figure 5:**
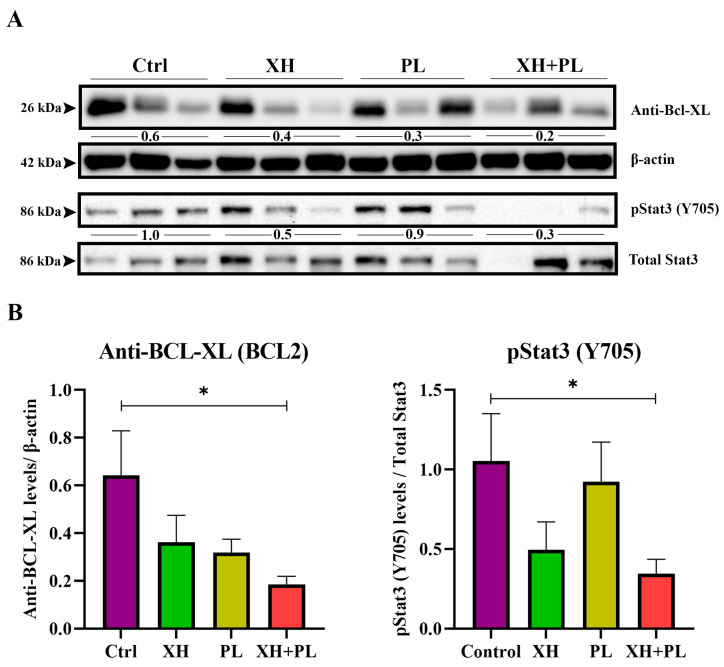
(**A**) Representative western blot of KPC mice tissues treated with vehicle (Control), xanthohumol (XH), plumbagin (PL), and a xanthohumol and plumbagin combination (XH/PL) [six mice were used per each treatment group] and probed for Anti-BCL-XL (BCL-2) and pStat3 Y705. β-actin was used as the loading control for BCL-2 and Total Stat3 for pStat3 (Y705). (**B**) Averaged quantification of Anti-BCL-XL and pStat3 (Y705) western blot signals normalised to their loading controls of the corresponding treatment groups. Data are means ± S.E. and an unpaired two-tailed student’s *t*-test was used for statistical analysis, * *p* < 0.01.

Frozen pancreatic tissues of mice treated with XH alone, PL alone, and a PL/XH combination were analysed by western blot to further corroborate the anticancer effect of this combination on proteins involved in the development of PDAC. A statistically significant reduction of the expression of the BCL2 protein in the mice group treated with PL/XH was found, compared to the expression of the vehicle-treated mice group (Figure 5 and Supplementary Figure S5). Similarly, pSTAT3 Y705 (Signal Transducer and Activator of Transcription 3, *STAT3*) levels in mice tissues were significantly reduced by both the treatment with single agents and the combination, while no significant decrease was observed for total STAT3 (Figure 5). The levels of CXCR4 could not be detected in mice tissues.

## 3. Discussion

**Figure 6:**
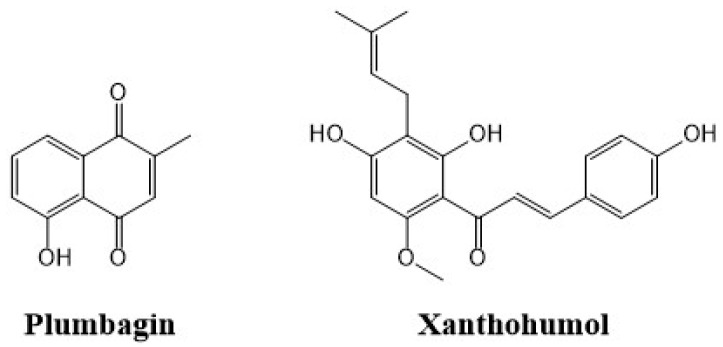
Chemical structures of plumbagin and xanthohumol.

The potential benefits of using natural plant extracts and plant-isolated compounds and their role in diminishing the risk of cancer have been widely reported [26,27,28]. In this study, after screening in vitro different plant extracts and isolated active compounds, we have focused on the most promising agents, PL, the active molecule from *Plumbago indica*, and XH, found in the inflorescences of *Humulus lupulus* (Figure 6).

When we tested PL and XH on pancreatic cancer models to examine the anticancer activity, the combination showed convincing results on different human pancreatic cancer cell lines. (Table 1). Interestingly, PL indicated a higher activity and the second lowest IC_50_ 0.53 µg/mL in AsPC-1 tumourspheres compared to the other cancer cell lines tested (Table 1). Pancreatic tumourspheres are cancer stem-like cells that are identified as a population of slow-cycling cells inside the tumour with self-renewal abilities [29]. Furthermore, these cells’ distinctive features are an increased level of tumourigenicity and a higher chemo-resistance [29]. Therefore, the above activity of PL on AsPC-1 tumourspheres is a promising discovery in this study. We subsequently tested the PL/XH combination on transgenic mouse pancreatic cancer cell lines (KPC and mT4-2D) and observed an increased anticancer activity on mT4-2D cells, with the lowest IC_50_ value (0.4 µg/mL) obtained from PL-treated cancer cell lines in the study (Table 1). Given the good results shown by PL in pancreatic cancer cell lines, we decided to test PL in cancer cell lines from the following other tumour types: human ovarian (A2780), human prostate (PC3), and breast adenocarcinoma (4T1) cell lines. In these cell lines, treatment with PL also produced low IC_50_ values, demonstrating good anticancer effects (Table 1). In addition, the anticancer activities of PL on prostate, pancreatic, breast, and lung cancer cell lines are also reported in the literature [30,31,32]. The IC_50_ values of XH on the human pancreatic cell lines HPAF-II and AsPC-1 were found to be 3.89 µg/mL and 6.8 µg/mL, respectively, showing XH to be relatively less effective compared with the IC_50_ values of PL. Furthermore, XH was found to have a stronger anticancer effect against the A2780 ovarian cancer cell lines, with an IC_50_ value of 0.3478 µg/mL (Table 1).

Chemo-resistance is becoming one of the main difficulties in cancer treatment [8,33]. Therefore, effective combination therapies are more reliable for the treatment of cancer. In this study, our focus was to perform combination treatments on pancreatic cancer cell lines to find a good and effective synergistic combination to progress to a transgenic model of pancreatic cancer. We observed a good synergistic effect after combining PL concentrations close to IC_25_ and IC_50_ with different concentrations of XH on both pancreatic cancer cell lines HPAF-II and mT4-2D that were tested. There is no data available in the literature for the synergism activity of PL with XH on any type of cancer.

More importantly, we showed that the PL and XH combination strikingly reduces tumour progression in an established and clinically relevant model of PDAC. To determine the mechanism of action of PL and XH treatments individually and in combination on pancreatic cancer cell lines, western blot analysis was performed on pancreatic cancer cell lines HPAF-II and mT4-2D. Results indicate that PL and XH both individually and in combination treatments target BCL2 protein on both cell lines. Moreover, based on the western blot images and quantification graphs, it is clearly demonstrated that both PL and XH combination treatments tested decreased the level of BCL2 protein compared to the control and compared to PL and XH treatments as single agents in both cell lines (Figure 2). BCL2 is known to be associated with apoptosis [34]. A clinical study showed that PL was able to regulate the intrinsic mitochondrial apoptotic pathway to induce cell apoptosis [35]. Furthermore, it was identified that the degradation of IκBα could be inhibited by PL and then suppressed the NF-κB activation and its translocation to the nucleus for function, which caused the downregulation of the anti-apoptosis protein BCL2 expression on various cancer cells [32,36,37]. The higher effect on BCL2 of the combination of PL and XH could also be attributed to a similar mechanism. Overexpression of CXCR4 in cancer cells is known to lead to tumour growth, invasion, angiogenesis, metastasis, relapse, and therapeutic resistance to cancer [38]. Therefore, CXCR4 becomes an important therapeutic target in the treatment of cancer [39]. Some studies identified PL as a major key player in downregulating the expression of the CXCR4 chemokine receptor in various types of cancers [40]. Consequently, identifying that PL and XH combinations could be able to downregulate CXCR4 more than PL alone can be considered a promising finding. Interestingly, a recent study that investigated the immunological consequences of inhibiting CXCR4 in PDAC patients who have historically resisted immunotherapy found that using continuous administration for one week of a small-molecule inhibitor of CXCR4 promotes an integrated immune response in metastatic lesions from these patients [41]. In addition, recent data also found that PL exerts a potent antitumour activity through the induction of anticancer immune response in non-small cell lung cancer models [23]. Therefore, it would be interesting to investigate in future studies the potential of the PL and XH combination in immunotherapy.

Our main data are obtained using a transgenic mouse model of pancreatic cancer (KPC) that is based upon the pancreatic-specific expression of endogenous mutant *Kras* and *Trp53* alleles. This model develops primary pancreatic tumours that precisely recapitulate the clinical, histopathologic, pharmacokinetic, and molecular features of human disease. In our study, we investigated the antitumour efficacy and the molecular mechanism of action of PL and XH in KPC mice survival. In addition, we have compared the activity of the combination to the gemcitabine and nab-paclitaxel combination, a treatment option for people with advanced pancreatic cancer. Our data show that the combination of gemcitabine and nab-paclitaxel gives a good response only in a portion of animals (as reported in clinical use) wherein it is not effective in most of them, whereas the PL/XH combination shows a good response in all animals. In addition, both PL and XH are used in clinical trials and therefore there is an immediate translational potential. Indeed, our search of available databases (ClinicalTrails.gov, accessed on 11 February 2024) found different ongoing clinical studies related to XH (NCT06225258, NCT05711212, NCT04590508, NCT03735420) and a synthetic form of PL (PCUR-101: NCT04677855, NCT03137758).

## 4. Materials and Methods

### 4.1. Extracts and Compounds Preparation

Plant extracts (ginger, chilli, pepper, peppermint, marjoram) were obtained from Flavex, Germany. The broccoli seed extract was obtained from CS Health (Louisville, KY, USA). The XH fraction of the hop extract was obtained from Hopsteiner (Au in der Hallertau, Germany). The active compounds (plumbagin, 6-gingerol, sulforaphane, 6-shogoal, capsaicin, zingerone, and piperine) were purchased from Sigma Aldrich (St. Louis, MO, USA). The pre-prepared powdered extracts were weighed and dissolved in dimethyl sulfoxide (DMSO) at the desired concentration (50 mg of solute per ml of DMSO for broccoli, broccoli and chilli, pepper, 10 mg/mL for ginger, peppermint, marjoram, hop extract, and XH). The mixture was filtered to obtain a clear solution that contained the components of interest. The active compound extracts, 6-gingerol, sulforaphane, 6-shogoal, capsaicin, zingerone, piperine, and PL, were dissolved in DMSO at a concentration of 10 mM; small 10 μL aliquots of the extracts and active compounds were prepared and frozen until needed. Each extract and compound was prepared specifically for the treatment, and after optimising concentrations, each treatment was undertaken 3 times on each cell line to achieve accurate results. Depending on the results obtained, the initial concentrations were modified, and combinations of compounds were tested on the cell lines. Subsequently, cells were treated with the most active extracts and compounds and protein analysis was undertaken by the western blotting technique.

### 4.2. Cell Culture

The mT4-2D cell line was a kind gift from Professor David Tuveson (CSHL) and was maintained in complete Dulbecco′s Modified Eagle′s Medium (DMEM) (Sigma, St. Louis, MO, USA) containing 10% foetal bovine serum (FBS) (Gibco, Waltham, MA, USA), 100 U/mL penicillin, 100 mg/mL streptomycin sulphate, and 2 mM L-glutamine [42]. The KPC (KrasLSL.G12D/+; p53R172H/+; PdxCretg/+) primary cell line, which we established as previously described [29], was cultured in standard cell culture vessels with DMEM supplemented with 10% FBS and added 1% Penicillin/Streptomycin. All other cancer cell lines were acquired from ATCC. HPAF-II was cultured in MEM medium (Sigma); AsPC-1, A2780, PC3, and 4T1 were cultured in RPMI 1640 medium (Sigma).

All the culture media, except for the cancer stem-like cell medium, were supplemented with 1% penicillin-streptomycin-glutamine (PSG) and 10% of FBS. Cancer stem-like cells were cultured in DMEM/F-12 medium (Sigma) supplemented as previously described and treated according to the protocol [29]. Cells were maintained in a humidified chamber (Nuaire DH auto flow CO_2_ air-jacketed incubator) at 37 °C with 5% CO_2_.

### 4.3. Cell Viability Assay

Cell viability assays were performed using a haemocytometer as described previously [43]. After seeding the cells for 24 h in 12 well plates, cells were treated with different plant extracts and pure compounds in a dose-response manner. After three independent repeats, results were examined and the IC_50_ for each extract and compound was calculated using the GraphPad Prism software 9.4.1.

### 4.4. MTT Assay

MTT assays were conducted using combination treatments of plant extracts and their compounds to examine the synergistic or antagonistic effect of the combinations. Thiazoyl Blue Tetrazolium Bromide (MTT) (Sigma) was prepared at 5 mg/mL in 1X PBS and was used for this assay. Approximately 5000 cells per well were seeded in 96 well tissue culture plates and treated with the combination treatments on the following day. After 72 h from the treatment, MTT (5 mg /mL, 10 µL per well) combined with 90 µL of media was added after the treatment was removed. Then, the plates were incubated at 37 °C for 2–4 h. After that, MTT was removed, and plates were allowed to dry for another 2 h. When fully dried, 70 µL of DMSO were added into each well and mixed gently using a shaker. At last, plates were read on a multimode plate reader (PerkinElmer, Waltham, MA, USA) at 570 nm. All data obtained were analysed using the CompuSyn software 1.0 by the Chou–Talalay method [24].

### 4.5. Determination of Combination Index and Dose Reduction Index

To study the effects of a combination treatment using PL and XH, cell viability was determined using the MTT assay after incubating cells with various concentrations of PL and XH in combination for 72 h. The Combination Index (CI) was calculated using the CompuSyn software 1.0 and following the classic isobologram equation: CI = D1/Dx1 + D2/Dx2, where Dx1 and Dx2 are the individual doses of PL and XH required to inhibit a given level of viability (x), and D1 and D2 are the doses of PL and XH required to inhibit the same level of viability (x) in combination. The CI values are then used to plot the CI versus Fraction Affected (FA) using GraphPad Prism software 9.4.1. The CI values help determine if the combination has an antagonistic effect (CI > 1) or a synergistic effect (CI < 1).

### 4.6. Western Blot Analysis

Western blot analysis was conducted to examine the expression patterns of cell apoptosis and cancer metastasis-regulating proteins (BCL2 and CXCR4) on pancreatic cancer cell lines treated with plant extracts and compounds. Tissues of mice treated with XH, PL, and combinations of the two were homogenised in RIPA buffer using pestle and mortar, sonicated and probed for BLC2, pERK, total ERK, pAkt 473, P-Akt 308, pStat3, and total Stat3 with western blot analysis, which was conducted according to the standard procedures as described previously [43]. All primary antibodies were used at 1:1000 dilution and incubated overnight at 4 °C with primary antibodies from Abcam: Anti-Bcl-XL (BCL2) (ab32370), Novus Biologicals CXCR4 (NB100-74396), Cell Signaling Technology^®^, Phospho-ERK (#4370), total ERK (#4695), Phospho-Akt 473 (#4060), Phospho-Akt (T308) (#4056), Phospho-Stat3 (Y705) (#9138), total Stat3 (#9139), and Actinin (#6487), the latter used as a loading control. The following day, membranes were incubated with HRP-conjugated secondary antibodies (Cell Signaling Technologies, Danvers, MA, USA) at a 1:40,000 dilution in 0.75% BSA in TBS/0.05% Tween-20 buffer for 1 h at room temperature. The signal was detected using the chemiluminescent detection reagent Amersham ECL PrimeWestern Blotting Detection Reagent (GE Healthcare Life Sciences, Chicago, IL, USA) imaged using ChemiDoc XRS+ (BioRad, Hercules, CA, USA), and quantified using Image Lab 5.1 software.

### 4.7. Animal Studies

All animal experiments were carried out in accordance with the National Health and Medical Research Council guidelines for the care and use of Laboratory animals (NHMRC, the Australian Code of Practice for the Care and Use of Animals for scientific purposes). All animal studies comply with the ARRIVE guidelines. The Curtin University Animal Ethics Committee approved procedures on KPC mice (AEC_2019_22). All animals were kept at 21 °C in ventilated cages cleaned weekly, with a 12 h light/12 h dark cycle and provided with water and food ad libitum. The sample size was estimated based on the power calculations performed previously in our group [44,45]. KRASWT/G12D, P53WT/R172H, PDX-1CRE+/+ (KPC) transgenic mice (both male and female), and control mice were maintained and genotyped (C57BL/6 genetic background) by the Animal Resources Centre (Murdoch, Western Australia) according to the original protocol [25]. Following the anticipated 80-day period, which was previously determined as the expected initiation of tumour development [44,45], KPC mice underwent daily palpation for the evaluation of tumour growth. After the tumours reached palpable size, animals were treated with PL (2 mg/kg), XH (40 mg/kg), and with the combination of two treatments or vehicle daily by intraperitoneal injections. Mice were assigned to the four arms (Vehicle, XH, PL, XH/PL) by simple randomisation by writing treatments on a piece of paper, folding, mixing, and then picking one by one. The animals underwent daily monitoring, and euthanasia was performed upon the detection of apparent signs of pain and distress, including substantial weight loss (exceeding 20% of the initial body weight), dehydration, ascites formation, breathing difficulties attributable to lymphoma progression, or signs of pain. Euthanasia was carried out by snipping the main cardiac vein, followed by organ perfusion through the heart. Mouse survival was depicted using a Kaplan–Meier curve and assessed using a log-rank test. 

### 4.8. Statistics

The results are all presented as mean ± SEM and are representative of at least three independent experiments. Statistical analyses were carried out using GraphPad Prism V9.4.1 software. Statistical significances were determined by performing two-tailed *t*-tests and one-way analysis of variance (ANOVA). *p*-value less than 0.05 is considered statistically significant. Kaplan–Meier curve was used to describe survival rates and its significance is quantified by log rank (Mantel–Cox) test.

## 5. Conclusions

In conclusion, we provide evidence of the pharmacological potential of the PL and XH combination that can block PDAC progression in vivo without discernible toxicity. Indeed, PL and XH individually displayed good anticancer activity in in vitro and in vivo studies and, interestingly, when used in combination, they demonstrated a much stronger effect compared to individual treatments, showing synergistic activity. Even more striking, a pancreatic cancer animal model treated with a combination of PL and XH resulted in a statistically significant increase in the survival of mice receiving this treatment compared to the controls. Western blot analysis of pancreatic cancer cell lines proved that the combination treatment affected the levels of BCL2, known to be associated with apoptosis and cancer metastasis, and in tissues from treated mice, a decrease in BCL2 and phospho-STAT3 (Y705) was found. Therefore, the combination of PL and XH demonstrated to be a promising candidate to be further validated as a potential anti-cancer treatment in clinical studies in the future.

## Figures and Tables

**Table 1:** IC_50_ values for each extract/compound determined with the cell viability assay after 72 h incubation in different cancer cell line (tumour source in brackets). IC_50_ was determined by a dose-response curve generated from at least five different inhibitor concentrations after three repeats using the GraphPad Prism software.

Extract/ Compound (Values in µg/mL)	Pancreas HPAF-II (H)	Pancreas ASPC-1 (H)	Tumour Type Ovary A2780 (H)	Prostate PC3 (H)	Pancreas mt4-2D (M)	Breast 4T1 (M)
**Broccoli seed**	184.2	237.1	228.5			
**Broccoli seed and Chilli**	207.3	245.7	250			592
**Ginger**	6.9	14.3	14.73	11.66		5.14
**Pepper**	22.42		28.33			
**Peppermint**		45.92				
**Marjoram**		41.22				
**Hop extract**	9.193	6.3	2.68	9.783		14.26
**6-shogaol**	4.22	4.94				
**Zingerone**		37.4				
**Piperine**		13.2				
**Capsaicin**	6.1	15.3				
**Sulforaphane**	1.46	1.32				
**Xanthohumol**	3.89	6.8	0.3478	4.968	9.15	1.26
**6-gingerol**	8.5	10.5				
**Plumbagin**	1.33	0.98	0.56	0.93	0.4	0.74

µg/mL = microgram per millilitre; Tumour source: H—human, M—mouse.

## Data Availability

The datasets generated during the current study are available from the corresponding author upon reasonable request.

## Supplementary Materials

The following supporting information can be downloaded at: https://www.mdpi.com/article/10.3390/ijms25042340/s1.
