# Supplementary material for: Synergistic Anticancer Activity of Plumbagin and Xanthohumol Combination on Pancreatic Cancer Models
Source: Int J Mol Sci. 2024 Feb 16;25(4):2340. doi: 10.3390/ijms25042340 (PMC10888694; doi:10.3390/ijms25042340)
Supplement: Supplementary file 1 [file ijms-25-02340-s001.zip › ijms-2861720-supplementary.pdf]

International Journal of *Molecular Sciences*

Supplementary Materials

Synergistic Anticancer Activity of Plumbagin and Xanthohumol Combination on Pancreatic Cancer Models

Ranjith Palanisamy, Nimnaka Indrajith Kahingalage, David Archibald, Ilaria Casari and Marco Falasca

HPAF II cell line

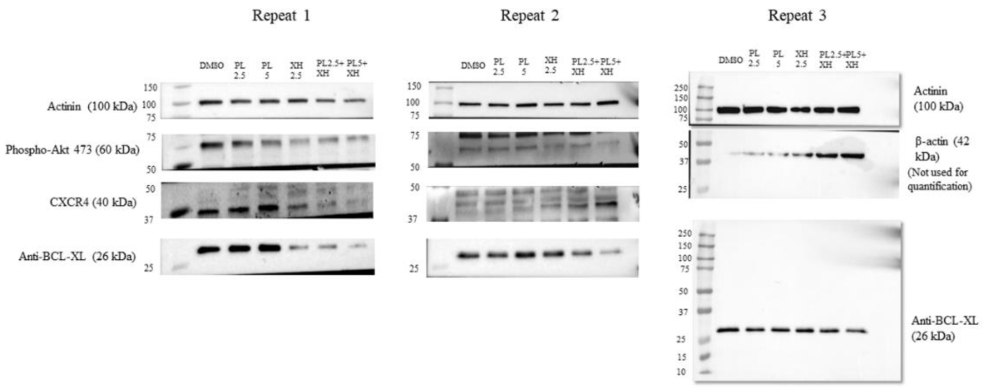

mT4-2D cell line

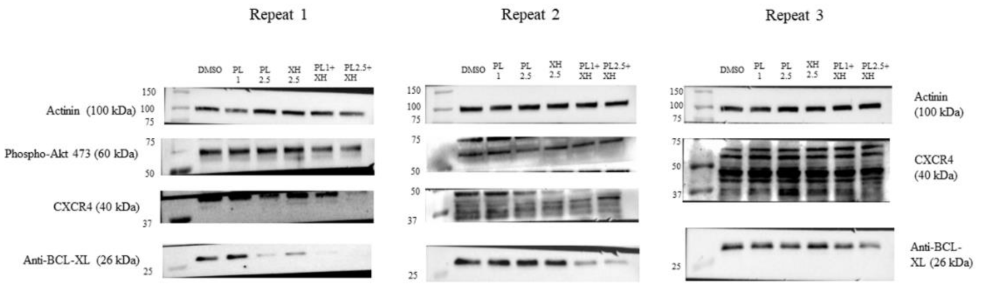

Supplementary Figure S1. Original western blots presented in Figure 2. Actinin and b-actin images for repeat three in HPAF-II cells have been obtained after stripping and reprobing western blotting membranes probed for anti-BCL-XL.

International Journal of Molecular Sciences

MDPI

A)

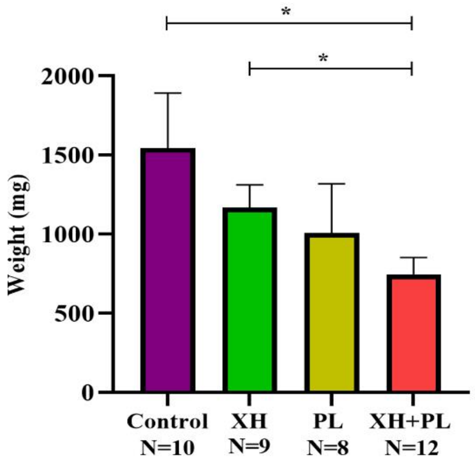

B)

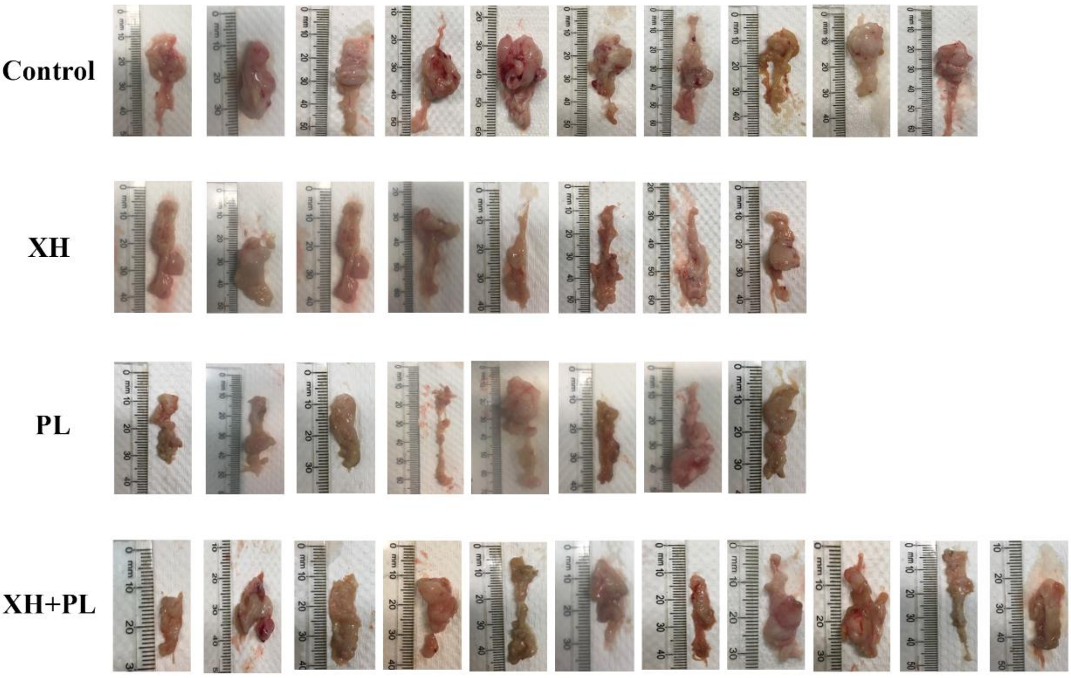

Supplementary Figure S2. (A) Graph compares resected pancreatic tumour weights from KPC mice treated with vehicle (N=10), xanthohumol (N=9), plumbagin (N=8) and xanthohumol+plumbagin (N=12). Data are represented as means ± S.E. and p values were calculated using unpaired two-tailed student's t-test, *$p < 0.01$. (B) Images of tumours obtained from all four treatment groups.

International Journal of
Molecular Sciences

MDPI

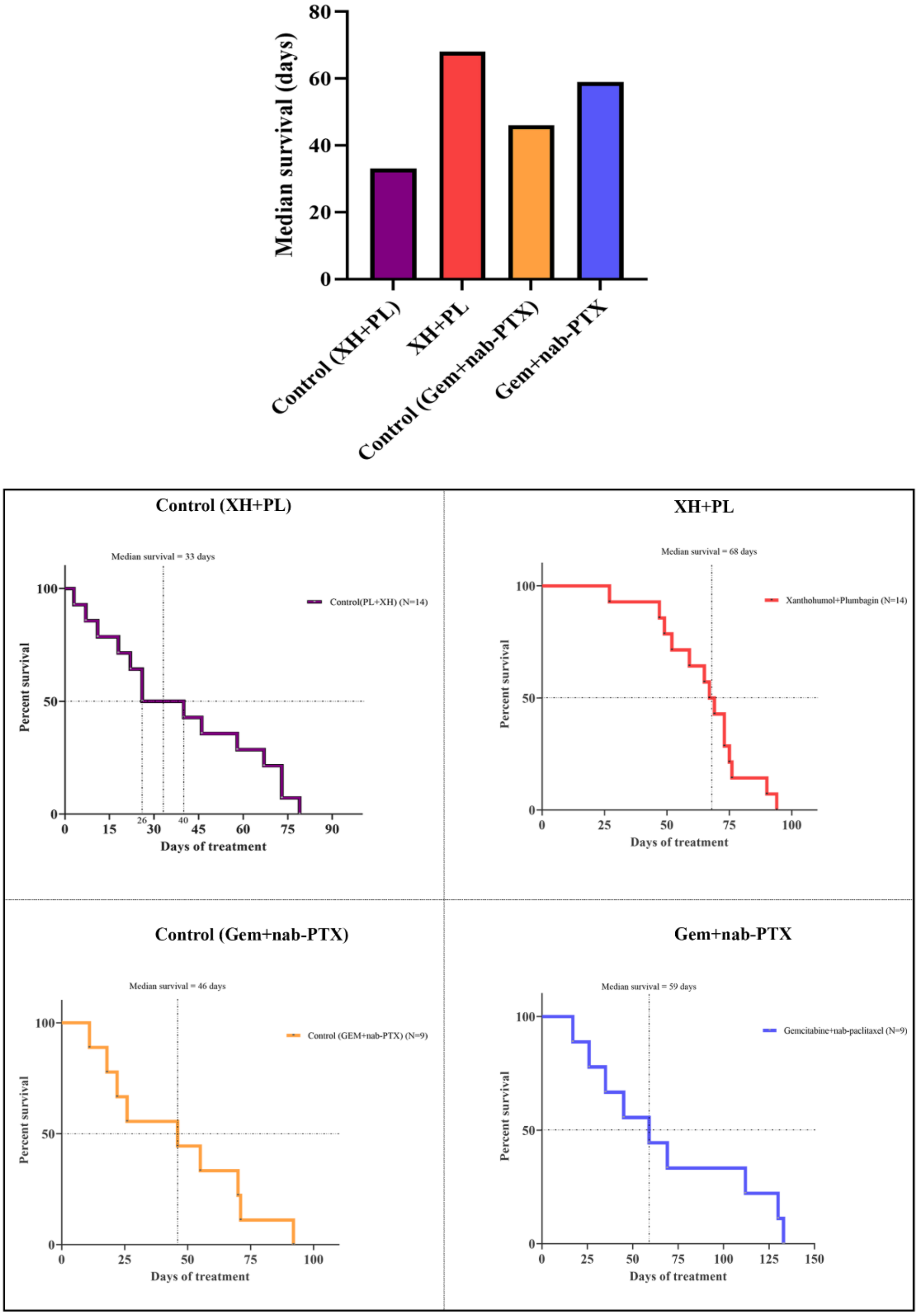

Supplementary Figure S3. The graph shows the median survival time obtained from the survival curves of xanthohumol plus plumbagin and its respective control (XH+PL), gemcitabine and nab-paclitaxel combination and its respective control (GEM+nab-PTX) in KPC mice. GraphPad Prism version 9.4.1 were used for statistical analysis. The images simulate the median survival time calculated by GraphPad Prism for all four treatment groups.

International Journal of
Molecular Sciences

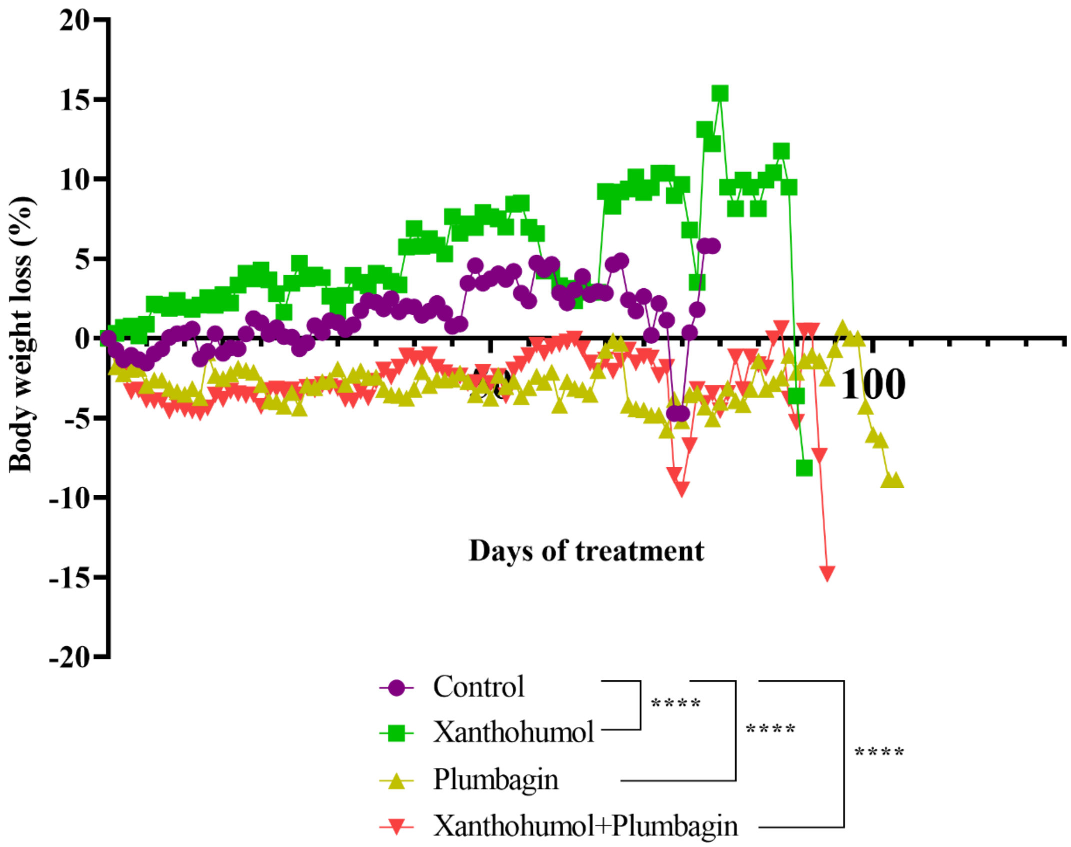

Supplementary Figure S4. Percentage of body weight loss of the KPC mice following treatment with plumbagin, xanthohumol, alone and in combination, and vehicle. Mice were weighed daily following the start of the treatment. Results are shown as means (n = 10-14 per group) and the data were analysed using one-way analysis of variance (ANOVA).

International Journal of Molecular Sciences

MDPI

Synergistic Anticancer Activity of Plumbagin and Xanthohumol Combination on Pancreatic Cancer Models

In vivo western blot repeats:

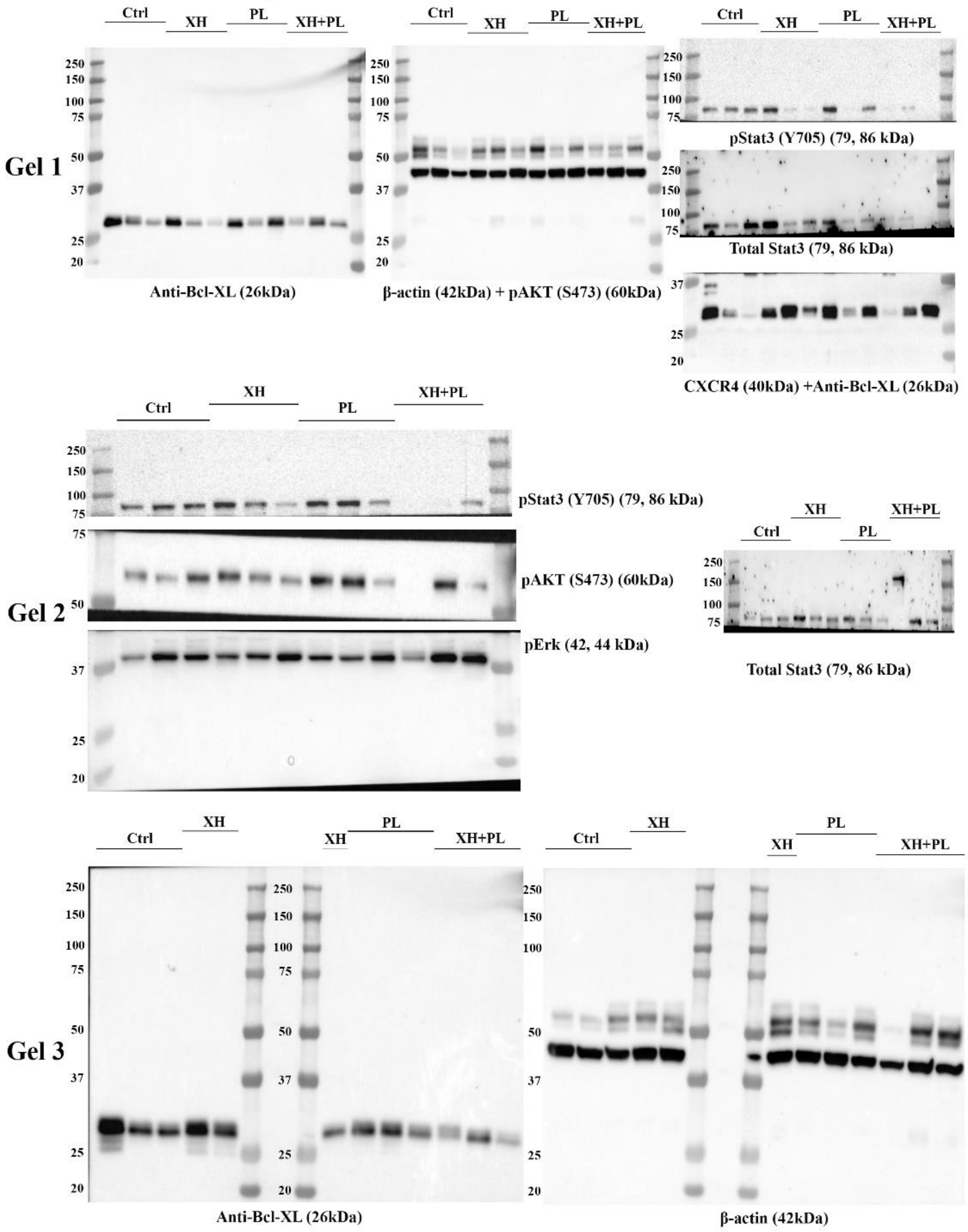

Supplementary Figure S5. Original western blots presented in Figure 5. Images on the right have been obtained after stripping and reprobing western blotting membranes probed in blots on the left.
